# Sensitivity Enhanced Photoacoustic Imaging Using a High-Frequency PZT Transducer with an Integrated Front-End Amplifier

**DOI:** 10.3390/s20030766

**Published:** 2020-01-30

**Authors:** Chen Yang, Xiaohua Jian, Xinle Zhu, Jiabing Lv, Yang Jiao, Zhile Han, Antonios Stylogiannis, Vasilis Ntziachristos, George Sergiadis, Yaoyao Cui

**Affiliations:** 1 University of Science and Technology of China, Hefei 230026, China; yangc@sibet.ac.cn; 2 Suzhou Institute of Biomedical Engineering and Technology, Chinese Academy of Sciences, Suzhou 215163, China; jianxh@sibet.ac.cn (X.J.); zhuxl@sibet.ac.cn (X.Z.); lvjb@sibet.ac.cn (J.L.); jiaoy@sibet.ac.cn (Y.J.); hanzl@sibet.ac.cn (Z.H.); sergiadi@sibet.ac.cn (G.S.); 3 Institute of Biological and Medical Imaging, Helmholtz Zentrum München, 85764 Neuherberg, Germany; stylogiannis@helmholtz-muenchen.de (A.S.); v.ntziachristos@helmholtz-muenchen.de (V.N.); 4 Chair for Biological Imaging, Technische Universität München, 81675 Munich, Germany; 5 School of Electrical and Computer Engineering, Aristotle University, 54124 Thessaloniki, Greece

**Keywords:** photoacoustic imaging, detection sensitivity, PZT transducer, front-end amplifier

## Abstract

Photoacoustic (PA) imaging is a hybrid imaging technique that can provide both structural and functional information of biological tissues. Due to limited permissible laser energy deposited on tissues, highly sensitive PA imaging is required. Here, we developed a 20 MHz lead zirconium titanate (PZT) transducer (1.5 mm × 3 mm) with front-end amplifier circuits for local signal processing to achieve sensitivity enhanced PA imaging. The electrical and acoustic performance was characterized. Experiments on phantoms and chicken breast tissue were conducted to validate the imaging performance. The fabricated prototype shows a bandwidth of 63% and achieves a noise equivalent pressure (NEP) of 0.24 mPa/√Hz and a receiving sensitivity of 62.1 μV/Pa at 20 MHz without degradation of the bandwidth. PA imaging of wire phantoms demonstrates that the prototype is capable of improving the detection sensitivity by 10 dB compared with the traditional transducer without integrated amplifier. In addition, in vitro experiments on chicken breast tissue show that structures could be imaged with enhanced contrast using the prototype and the imaging depth range was improved by 1 mm. These results demonstrate that the transducer with an integrated front-end amplifier enables highly sensitive PA imaging with improved penetration depth. The proposed method holds the potential for visualization of deep tissue structures and enhanced detection of weak physiological changes.

## 1. Introduction

Photoacoustic (PA) imaging has drawn much attention worldwide during the last two decades with its compelling advantage of combining the high-contrast of pure optical imaging with the deep penetration depth of ultrasound (US) imaging [1,2,3,4,5]. Different from the conventional pulse-echo based US imaging, PA imaging is typically performed by illuminating the target with a nanosecond laser pulse and then detecting the ultrasonic signals using a mechanically scanned single-element ultrasonic transducer or an array probe [6,7,8]. PA imaging has been demonstrated to be capable of high resolution structural imaging such as mapping of subcutaneous micro-vessels [9,10,11,12,13], anatomic features discrimination [14,15,16], and detection of atherosclerotic plaques based on the lipid-specific contrast [17,18,19]. Meanwhile, PA imaging also shows the potential for physiological information measurements such as hemoglobin oxygen saturation (sO2) [20,21,22], metabolic oxygen rate [23], and tissue temperature distribution [24,25,26]. With the help of exogenous contrasts, PA molecular imaging and gene imaging has been achieved [27,28,29,30,31].

While PA imaging has been explored in a variety of biomedical applications, there is an extensive demand to improve the detection sensitivity [32,33,34]. Since the signal attenuation caused by the scattering of light and the attenuation of the acoustic waves is more than one order of magnitude per centimeter at 10 MHz [35], the acoustic pressure of the PA signals arriving at the transducer is in the Pa or sub-Pa range depending on the depth [36]. When high frequency transducers (≥ 20 MHz) are used to achieve high spatial resolutions, signals become extremely weak considering the frequency-dependent attenuation on the order of 0.5 dB∙cm^-1^∙MHz^-1^ in biological tissues [37]. Thus, high detection sensitivity is required in PA imaging. For a given absorption contrast, the detection sensitivity of PA imaging mainly depends on the incident light exposure and the efficiency of the detector [38]. As the laser energy increases, the enhancement of PA signal is eventually limited by the optical absorption saturation and the maximum permissible exposure specified by American National Standards Institute (ANSI) [39]. Therefore, highly sensitive acoustic detectors are of particular interest.

Optical and interferometric detectors like Fabry-Perot interferometers, are frequently utilized in PA imaging for all-optical light delivery and high sensitivity detection. However, they are difficult to form an array for fast imaging. Capacitive micromachined ultrasonic transducers (CMUTs) are also used for PA imaging because of their attractive large bandwidth and ease of miniaturization, but they are not readily available and a large DC bias is required to achieve high performance [40]. The most widely used detectors in PA imaging are the lead zirconium titanate (PZT) transducers due to their well-established technology, low cost of implementation, and high sensitivity [41]. However, there is a trade-off between the size and the sensitivity of the PZT transducer. The achieved sensitivity usually drops with reduced aperture and gets further degraded by the connecting cable, especially for high frequency transducers [42].

Integrating ultrasound transducer with front-end circuits is an efficient way to process signals locally and maintain the signal integrity. Transducer arrays with custom application-specified integrated circuits (ASIC) have been previously demonstrated in 3D ultrasound imaging [42,43,44]. However, these devices overwhelmingly operate with frequencies lower than 10 MHz, which are not enough for detecting the high frequency components of the PA signals. Their detection sensitivity also still requires further optimization for PA imaging. In addition, PA microscopic applications generally demand single-element high-frequency transducers with high sensitivity to detect signals generated by tiny biological structures [45].

In this work, we proposed a 20 MHz PZT transducer with an integrated front-end amplifier for PA imaging with high sensitivity. The amplifier has an ultra-wide bandwidth beyond 200 MHz and is capable of offering front-end signal amplification with a low noise figure while keeping the transducer sufficiently compact. The design, fabrication, and characterization of a prototype are presented in this work. Using the transducer with an integrated front-end amplifier, PA imaging experiments were performed on phantoms and chicken breast tissue in vitro to demonstrate the improvement of the signal-to-noise ratio (SNR) and the imaging depth range. High detection sensitivity achieved by the integrated transducer may enable a variety of pre-clinical and clinical applications that requires further in vivo evaluation. A preliminary version of this work has been reported [46].

## 2. Materials and Methods

### 2.1. PZT Transducer with an Integrated Front-End Amplifier

**Figure 1:**
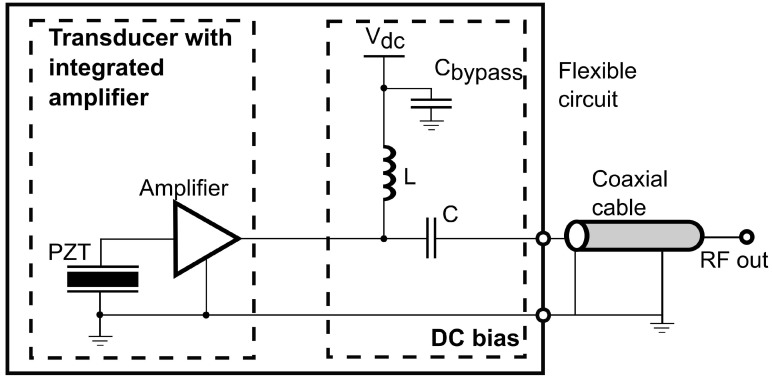
Circuit diagram of the integrated front-end amplifier circuits.

The front-end amplifier used is designed as a rectangular die with a highly compact structure such that simple auxiliary circuits are required for operation, as shown in Figure 1. Similar to typical implementation [47], only a bias tee composed of an inductor L and a capacitor C is necessary for DC bias of the amplifier and AC coupling to the coaxial cable.

A front-end amplifier closely coupled to the PZT element firstly reduces the additional noise at the input of the amplifier by minimizing the electrical path between the transducer and the amplifier. Since the transmission line will introduce noise and interference to the signal, it is always crucial to place the amplifier to the transducer as close as possible to obtain a clean signal. Secondly, the front-end amplifier offers a high gain with a low noise figure to generate a strong enough signal to be noise-tolerant, such that the noise added by the following transmission lines will not significantly degrade the SNR.

**Figure 2:**
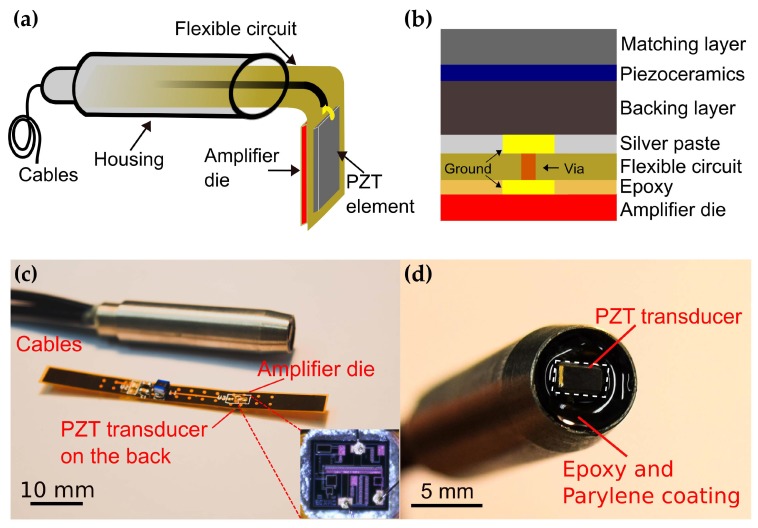
Schematic of the assembly of the transducer (**a**) and the cross section of the PZT transducer with an integrated front-end amplifier (**b**). (**c**) Photo of the fabricated prototype and the flexible circuit. Inset: photo of the amplifier die and bonding wires (**d**) PZT transducer on the tip.

The assembly of the proposed PZT transducer with an integrated amplifier die is illustrated in Figure 2a. A 0.3-mm thick flexible circuit provides the electrical connections for biasing and signal handling and serves as an intermediate connector between the transducer and the die. For proper grounding, the die was attached on a flat gold-coated plate on the rear side of the flexible circuit. The bond pads on the die were wire-bonded to the corresponding pads on the flexible circuit with 1-mil diameter gold wires. The miniature feature of the die (820 μm × 760 μm) enables tight integration with transducers of various sizes. An unfocused PZT transducer with an active area of 1.5 mm × 3 mm was built in house. The size of the transducer was determined to achieve optimal sensitivity and ease of fabrication. The fabricated PZT transducer was attached on the front side of the flexible circuit using conductive silver paste (H20E, EpoTek, Billerica, MA, USA). The cross section of the transducer with an integrated amplifier die is shown in Figure 2b.

A cooper plated via was drilled through the flexible circuit to connect the ground of the transducer and the die mounting plate. The top electrode of the transducer was interfaced to the input pad of the die by wire bonding. All bonding wires were kept as short as possible to reduce performance degradation caused by series inductance. Two 1-m long coaxial cables for signal output and DC power supply respectively, were soldered to the corresponding pads on the flexible circuit. Then the flexible circuit was inserted into a customized housing and fixed with an epoxy to reduce the interference from outside. After successful assembly of the transducer, a 14-μm parylene film (Parylene C, Special Coating Systems, Indianapolis, IN, USA) was coated on the transducer for acoustic impedance matching and electrical isolation. The fabricated prototype is shown in Figure 2c,d. For comparison, another PZT transducer of the same aperture and assembly but without the integrated amplifier was fabricated. In the rest of the paper, we referred to the transducer with and without an integrated amplifier as aUST and UST, respectively.

### 2.2. Transducer Characterization

Before being attached on the flexible circuit, the electrical impedance of the fabricated PZT element was measured using an impedance analyzer (E991A, Keysight Technologies, Santa Rosa, CA, USA). The output frequency response of the proposed circuit shown in Figure 1 was measured using a network analyzer (E5061B, Keysight Technologies, Santa Rosa, CA, USA). The signal source was applied to the input of the amplifier via a capacitor with an impedance comparable to that of the fabricated PZT element. The output noise spectrum of the transducer with an integrated amplifier was achieved by computing the power spectral density of the measured noise.

**Figure 3:**
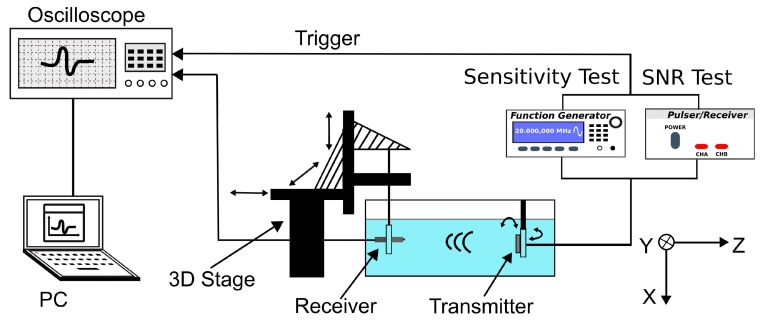
Schematic of the experimental setup of the receiving test. The transmitter is driven by the function generator during the sensitivity test and by the pulser–receiver during the SNR test.

To characterize the receiving sensitivity of the fabricated transducer, a 20 MHz unfocused transducer was used as a transmitter and was driven by a function generator (33250A, Keysight Technologies, Santa Rosa, CA, USA) to transmit acoustic waves, as shown in Figure 3. The UST and the aUST were mounted on a 3D stage and positioned at ~3 mm away from the transmitter to receive the acoustic signals, respectively. The output signals were averaged 16 times and recorded by an oscilloscope (DPO5034, Tektronix, Beaverton, OR, USA) for offline analysis. To calibrate the acoustic pressure of the transmitted waves, a PVDF needle hydrophone with a diameter of 0.2 mm (Precision Acoustics, Dorchester, UK) was placed at the same position after the tests. The output of the function generator was a 10-cycle 5-Vpp burst waveform, the frequency of which was swept from 10 to 30 MHz in a step of 1 MHz because the hydrophone used is only calibrated at these frequencies.

To further evaluate the receiving SNR of the transducer, the transmitter was driven by a negative voltage pulse of 5-ns pulse width and 150-V peak amplitude generated by a pulser/receiver (DPR500, Imaginant). The UST and the aUST were setup to detect the pulse waves at different positions along the beam axis. The SNR, which is defined as the ratio of the peak amplitude of the signal to the root-mean-square value of the noise, was computed using MATLAB (The MathWorks, Natick, MA, USA).

### 2.3. System Configuration

**Figure 4:**
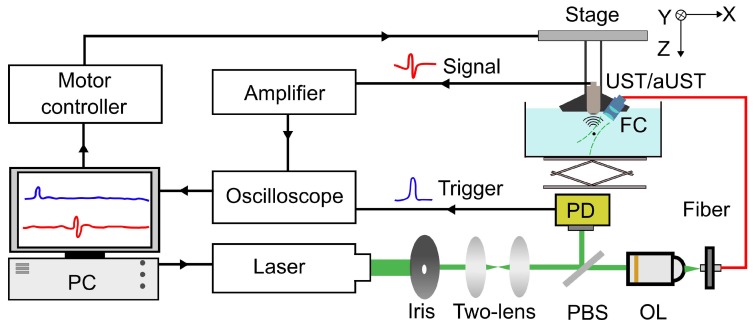
Schematic of the photoacoustic (PA) imaging system. PBS, pellicle beam splitter; OL, objective lens; PD, photodiode; FC, fiber collimator; UST: transducer without an integrated amplifier and aUST: transducer with an integrated amplifier.

A PA imaging system was developed to evaluate the imaging performance of the prototype, as shown in Figure 4. An optical parametric oscillator (OPO) laser (VIBRANT 532, OPOTEK, Carlsbad, CA, USA) with a tunable output was used to irradiate the sample with a laser pulse of 10-ns at a wavelength of 800 nm and a repetition rate of 10 Hz. The laser beam was first reshaped by an iris (ID25, Thorlabs, Newton, NJ, USA) before being collimated by a double-lens system. The collimated laser beam was split into two beams via a pellicle beam splitter (BP108, Thorlabs, Newton, NJ, USA). One of them was directed to a fast photodiode (DET10A2, Thorlabs, Newton, NJ, USA) for monitoring the laser stability and triggering the oscilloscope. Another one was coupled to a multi-mode fiber (CSH1000, Shenzhen Xinrui Optical, Shenzhen, China) with a 1-mm core diameter using a microscope objective (RMS10X, Olympus, Tokyo, Japan). The output light from the fiber was focused on the sample by a fiber collimator (F220SMA-780, Thorlabs, Newton, NJ, USA) and an aspheric lens (A260TM-A, Thorlabs, Newton, NJ, USA), resulting in an illumination spot with a diameter of ~1.4 mm. The generated PA signals were detected by the UST and the aUST respectively, amplified by an amplifier with 55 dB gain (CLC-10K0.5G-5510-S, Connphy, Baltimore, MD, USA), and captured using an oscilloscope (MDO3022, Tektronix, Beaverton, OR, USA). An imaging head composed of the fiber collimator and the transducer was mounted on a linear stage (SC3002B, Zolix, Beijing, China) for imaging. To acquire a B-scan image, the imaging head was scanned along the X-axis with a step size of 100 µm. The whole imaging process including the move of the stage, the emitting of the laser and the data acquisition, was controlled by MATLAB software.

### 2.4. Phantom Preparation

A 0.5-mm pencil lead fixed on a mounting plate using UV (Ultraviolet) gel was used to demonstrate the enhanced detection of PA signals using the transducer with an integrated front-end amplifier. Two wire phantoms were prepared for evaluation of the spatial resolutions and the imaging depth range. The first wire phantom consisted of five 50-μm tungsten wires attached on a U-shape holder. The wires were aligned in parallel with the Y-axis and spaced ~0.5 mm along the Z-axis. The second wire phantom had a 30-μm and a 50-μm tungsten wire crossing each other in the XZ plane. All wires were immersed in water for ultrasonic coupling. In vitro experiments were performed on a chicken breast tissue phantom with five 200-μm wires embedded under the skin surface at depths ranging from 0.2 to 0.9 mm.

### 2.5. Data Processing

PA signals emitted from samples were digitized at a sampling rate of 100 MS/s and averaged 8 times before being stored in a PC. The number of averaging was decreased compared to the before-mentioned 16 times averaging to reduce the acquisition time. The data were analyzed offline using MATLAB. Prior to image reconstruction, a band-pass filter with cut-off frequencies between 2 and 35 MHz was applied to the original signals to eliminate out-of-band noise and aliasing. Then, the time-resolved PA signals were converted to depth-resolved A-lines based on the time of flight (ToF) principle. To make the image finer and improve the accuracy of Gauss fitting, each A-line was interpolated by a factor of 5 corresponding to 500 MS/s sampling rate. A B-scan image was reconstructed by stacking Hilbert transformed A-lines along the scan direction.

## 3. Results and Discussion

### 3.1. Transducer Characterization

**Figure 5:**
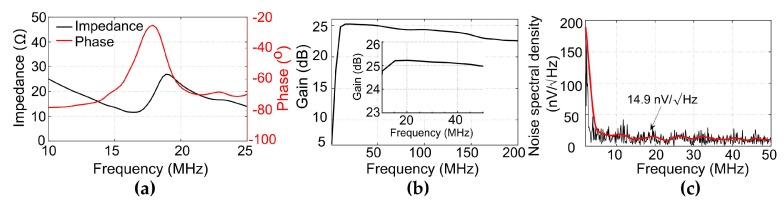
(**a**) Electrical impedance of the PZT transducer. (**b**) Output frequency response of the amplifier circuits. Inset: Output frequency response from 10 to 50 MHz. (**c**) Output noise spectral density of the transducer with an integrated amplifier.

Figure 5a shows the electrical impedance of the PZT element. The center frequency was measured to be 18 MHz. Figure 5b shows the measured output frequency response of the front-end amplifier circuits. The measured −3 dB bandwidth ranges from 5 to 200 MHz, making the amplifier suitable for detection of the wideband PA signals. Notably, the amplifier shows a flat response of over 25 dB gain from 10 to 50 MHz, which can completely cover the bandwidth of the fabricated PZT transducer. Figure 5c shows the output noise voltage spectrum of the transducer with an integrated amplifier. The noise density at 20 MHz was 14.9 nV/√Hz and the integrated noise level from 2 to 35 MHz was 138 µV.

**Figure 6:**
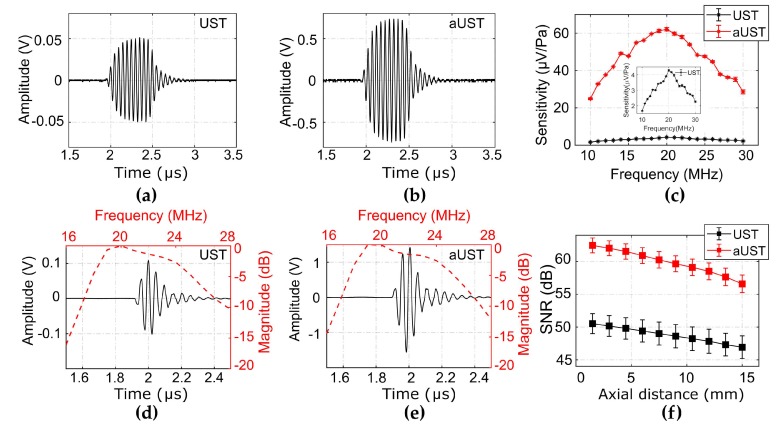
Performance characterization. (**a**–**b**) Typical received signals under a 10-cycle burst at 20 MHz using the UST and the aUST, respectively. (**c**) Calibrated sensitivity. Insert: sensitivity of the UST. (**d**–**e**) Typical pulse signals received at ~3 mm using the UST and the aUST, respectively. Red line: frequency spectrum; black line: pulse signal. (**f**) Signal-to-noise ratios (SNRs) along the beam axis. The graphs show results from three separate tests and the data are shown as means ± SD. UST: transducer without integrated amplifier and aUST: transducer with an integrated amplifier.

Figure 6a,b presents the typical received signals under a 10-cycle burst at 20 MHz using the UST and the aUST, respectively. The peak-to-peak amplitude of the signal detected by the aUST was about 15-fold higher than that detected by the UST. The amplification of the signal shows an agreement with the frequency response of the amplification circuits mentioned before. By dividing the calibrated acoustic pressure by the amplitude of the detected signal, the receiving sensitivity across the bandwidth of 10 MHz to 30 MHz was obtained. As shown in Figure 6c, the aUST achieved a significantly enhanced sensitivity of 62.1 μV/Pa at 20 MHz compared with that of the UST (4.3 μV/Pa at 20 MHz). In comparison, Chen et al. demonstrated a AIN-based piezoelectric transducer with the sensitivity of 4.22 µV/Pa [48]. As previously stated, the noise equivalent pressure (NEP) is computed by dividing the output noise by the receiving sensitivity of the transducer [49]. According to the results shown in Figure 6c, the NEP of the aUST was determined to be 0.24 mPa/√Hz at 20 MHz.

Due to the flat response of the amplifier over the frequency range of 10–50 MHz, the −6 dB bandwidth and the center frequency of the signal detected by the aUST (Figure 6d) present no significant changes compared with that detected by the UST (Figure 6e). The receiving performance of both transducers is summarized in Table 1. It should be noted that the bandwidth mentioned here is not the detection bandwidth of the transducer under test, because the received signal is the convolution of the impulse response of the receiver and the transmitted signal. The SNRs of the received signals detected by the aUST were improved by at least 10 dB compared with that detected by the UST (Figure 6f). Both transducers achieved decreasing SNRs along the beam axis due to the divergence of the acoustic beam. As shown in Table 1, the NEP achieved by the aUST was ~9 dB lower than that achieved by the UST, which well explains the measured SNR improvement considering the imperfect alignment during the measurements. These results showcase that integrating front-end amplifier to the transducer is an effective method to improve the detection sensitivity of the acoustic detector while maintaining the bandwidth. The achieved high sensitivity will benefit PA signal detection under low energy depositions.

### 3.2. Enhanced Detection of the PA Signal

**Figure 7:**
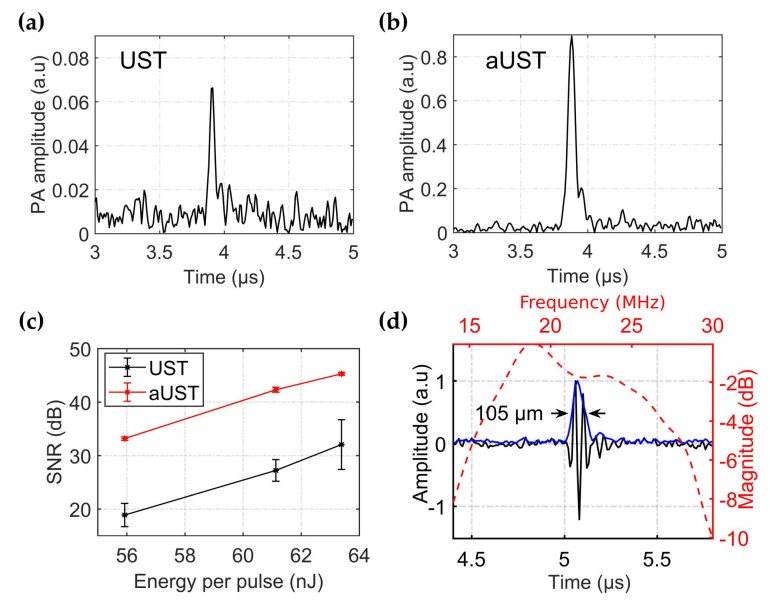
Enhanced detection of PA signals. (**a**–**b**) Envelope of the typical signal detected by the UST and the aUST respectively, under an optical fluence of ~56 nJ per pulse. The range of the amplitude axis in (**a**) is set to one tenth of that in (**b**) for better illustration. (**c**) Signal-to-noise ratios (SNRs) under different light fluences. The graphs show results from three separate tests and the data are shown as means ± SD. (**d**) Frequency response of the transducer with an integrated amplifier. UST: transducer without integrated amplifier and aUST: transducer with an integrated amplifier.

To evaluate the enhancement of detected PA signals, the pencil lead was illuminated under different light fluences and the generated PA signals were received using the UST and the aUST, separately. When switching from the UST to the aUST, the experimental configurations, including the laser energy and the position of the phantom, remained the same. The depth position of the aUST was adjusted until the detected PA signals had the same flight time to the UST. Therefore, it could be assured that the two transducers were placed in the same position during the measurements. The output energy of the laser was adjusted by changing the Q-switch delay and attenuated by the iris. To compensate for the fluctuation of the laser output, the amplitude of the PA signal was corrected by the laser energy per pulse. Figure 7a,b present the typical PA signals detected by the UST and the aUST under 56 nJ per pulse. The amplitude of the PA signal is enhanced by approximately 14-fold using the aUST, which accords well with the results shown in Figure 6. In comparison with the UST, the aUST improved the SNR by ~10 dB under different laser energies (Figure 7c). When the light fluence decreased, the aUST still showed satisfying SNR because of the enhanced detection sensitivity. The receiving bandwidth of the transducer with an integrated amplifier was measured using a broadband PA signal generated from a 10-µm wire. According to Figure 7d, the transducer had a −6 dB fractional bandwidth of 63%. The full width at half magnitude (FWHM) of the Hilbert transformed envelope of the PA signal was measured to be 105 µm based on the sound velocity of 1500 m/s in water, which corresponds to the axial resolution of the transducer.

### 3.3. Imaging of Wire Phantoms

**Figure 8:**
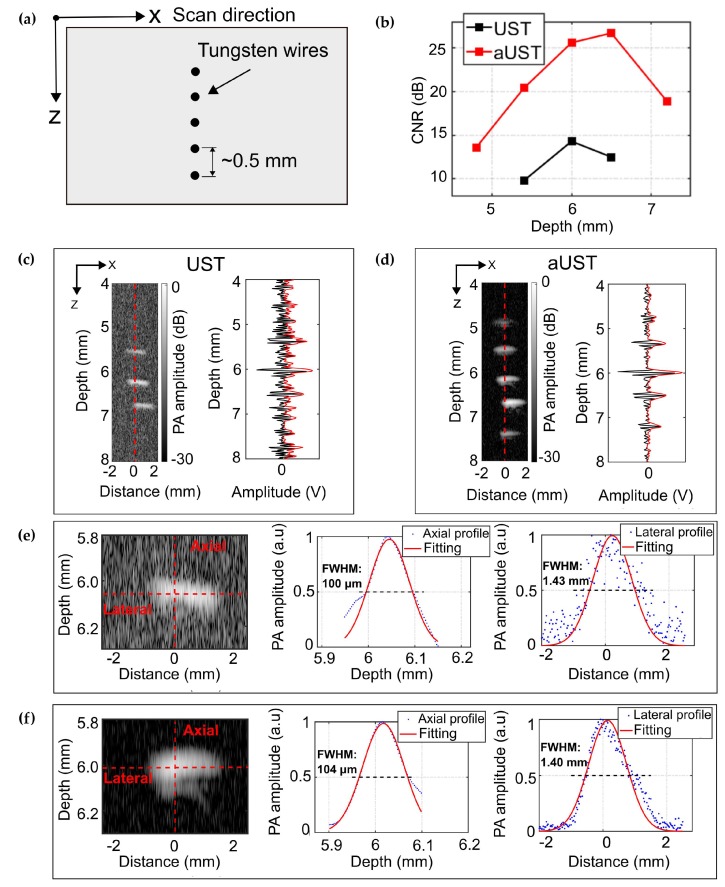
PA imaging of five tungsten wires. (**a**) Schematic of the alignment of the wires. (**b**) Contrast-to-noise ratio (CNR) along depth. (**c**) B-scan image of five tungsten wires acquired by the UST at 30 dB dynamic range and the PA signal along the red dashed line. (**d**) B-scan image of five tungsten wires acquired by the aUST at 30 dB dynamic range and the PA signal along the red dashed line. (**d**,**e**) Axial and lateral resolution at a representative depth of 6 mm by the UST. (**f**) Axial and lateral resolution at a representative depth of 6 mm by the aUST. UST: transducer without integrated amplifier and aUST: transducer with an integrated amplifier.

We evaluated the spatial resolution and the imaging depth range of the fabricated prototype using a phantom containing five 50-µm tungsten wires aligned in a depth range of ~2.5 mm with spacings of ~0.5 mm, as shown in Figure 8a. For comparison, the same area was imaged using the UST. Figure 8c presents the B-scan image acquired by the UST. Three wires located within the center of the illumination spot are clearly shown on the image, while the other two out-of-focus wires cannot be observed. Since a focused light was used in our system to illuminate the sample, wires placed in the optical focus absorb more laser energy and generate stronger signals than the wires away from the focus. This is further explained by the Hilbert-transformed envelope of the PA signal along the red dashed line in the B-scan image. Signals emitted from the central three wires could be well discriminated, while signals generated from other two wires were emerged in the noise floor. Compared with the UST, the aUST was able to detect all five wires with improved contrast (Figure 8d), corresponding to an enlarged imaging depth range of ~2.5 mm. The contrast-to-noise ratio (CNR) of the B-scan image was calculated as the ratio of the difference between the mean amplitude of the signal and the noise, to the root-mean-square value of the background noise area taken from the image [50]. Figure 8b presents the CNRs at five depths from 4.8 to 7.3 mm. There was a significant improvement of the CNR by more than 10 dB obtained by the aUST. Since the sample was illuminated with an angle of 45° and the focus was placed ~6 mm under the transducer, the CNR peaks at ~6 mm depth and decreased as the wires were away from the optical focus. The slight differences between the graphs of the UST and the aUST were attributed to the non-identical directivity of the transducer and the slight position deviations during scanning.

The wire reconstructed at ~6 mm was used to estimate the spatial resolutions. Gaussian fitted profiles of the Hilbert transformed PA envelopes were computed using the Curve Fitting Toolbox in MATLAB. The resolution is defined as the FWHM of the fitted curve. As illustrated in Figure 8f, the axial resolution of the aUST was measured to be 104 μm, which is in good agreement with the result shown in Figure 7d. Both transducers achieve comparable axial resolutions because the integrated amplifier did not change the bandwidth significantly, as summarized in Table 1. The lateral resolution of the current system was mainly dominated by the size of the optical beam at the focal plane, since the sensitivity field of the unfocused transducer was larger than the optical focus. To better show the improvement of the imaging depth range, we used a multi-mode fiber to achieve a large illumination spot of 1.4 mm in diameter.

**Figure 9:**
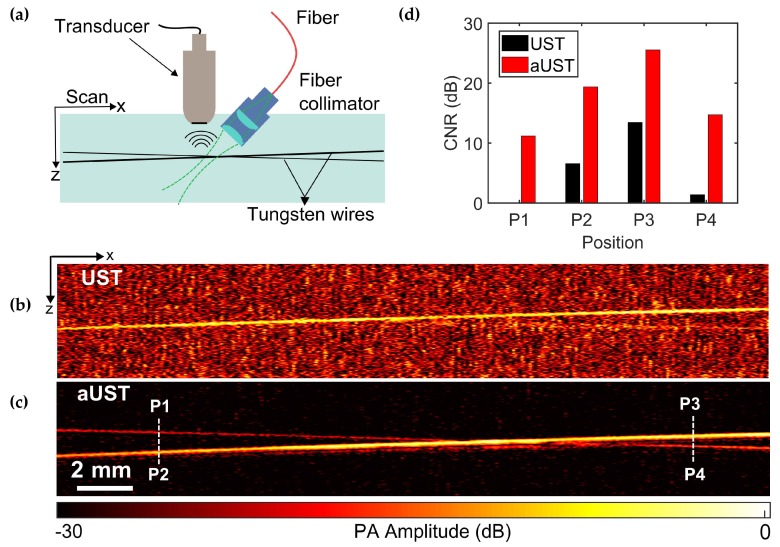
PA imaging of two crossing wires. (**a**) Schematic of the alignment of the transducer, the light source and the phantom. (**b**) B-scan image of two crossing wires acquired by the UST at 25 dB dynamic range. (**c**) B-scan image of two crossing wires acquired by the aUST at 30 dB dynamic range. (**d**) Contrast-to-noise ratios (CNRs) at four regions labeled by P1, P2, P3, and P4 in (**c**). The CNR of the UST at P1 is not available. UST: transducer without integrated amplifier and aUST: transducer with an integrated amplifier.

**Figure 10:**
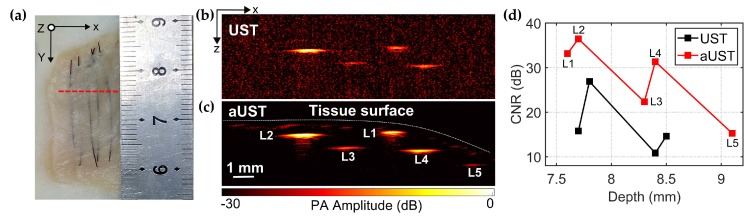
PA imaging of the chicken breast tissue phantom. (**a**) Photo of the chicken breast tissue phantom. Red dashed line represents the scanning plane. (**b**) B-scan image acquired by the UST at a 30 dB dynamic range. (**c**) B-scan image acquired by the aUST at a 30 dB dynamic range. (**d**) Contrast-to-noise ratios (CNRs) at different depths. CNR of UST at ~9.1 mm is not available. UST: transducer without an integrated amplifier and aUST: transducer with an integrated amplifier.

To further demonstrate the imaging performance of the transducer, a wire phantom consisting of two crossing wires with diameters of 30 μm and 50 μm was imaged using the UST and the aUST, respectively. Figure 9a illustrates the experimental setup. The angle between two wires was small enough to assure that both wires were within the light spot. Due to low optical absorption, PA signals emitted from the 30-μm wire were too weak to be detected by the UST, thus the 30-μm wire could be barely identified in the B-scan image, as shown in Figure 9b. In comparison, both wires were clearly observed in Figure 9c with superior contrast, indicating the improved detection sensitivity of the aUST. The crossing point was not clearly distinguished because of two reasons: firstly, signals from two wires are strongly interfered when they get closer, resulting in an overlapped Hilbert transformed PA envelope and secondly, the axial resolution of the system is measured to be ~104 μm, thus when the distance of two wires comes to this range, they cannot be resolved. A possible solution to this problem is to use a transducer of higher frequency and broader bandwidth to achieve finer axial resolution. Figure 9d presents the CNRs at four locations labeled by P1-P4 in Figure 10c. The CNR of the UST at P1 is not available because the wire at this position cannot be recognized. In comparison, the CNRs were improved by ~10 dB using the aUST, which agreed well with the before-mentioned results. The results of wire phantoms imaging experiments demonstrated that the transducer with an integrated front-end amplifier was capable of improving the image quality and depth of PA imaging by offering enhanced detection sensitivity.

### 3.4. Imaging of Chicken Breast Tissue

We also conducted in vitro experiments on chicken breast tissue phantom with five 200-μm tungsten wires embedded under the tissue surface to further validate the imaging performance. For this experiment, the output laser energy from the fiber was limited to 850 nJ per pulse, thus the corresponding energy density deposited at the focus is calculated to be ~55 μJ/cm^2^, which is far lower than the laser safety limit for skin exposure (32 mJ/cm^2^ at 800 nm) outlined by ANSI [39]. We limited the laser energy to demonstrate that the prototype is capable of providing high CNR and deep imaging depth even at such low light fluence at which the conventional UST cannot obtain satisfying images.

Figure 10a shows the photo of the chicken breast tissue phantom and the red dashed line represents the scanning slice. Figure 10b,c present the B-scan images acquired by the UST and the aUST, respectively. In Figure 10c, all five wires, including L5 that is not shown in Figure 10b, were clearly visualized with high contrast and the distribution of the tissue surface could be well recognized. The distances of the embedded wires L1–L5 from the tissue surface were estimated to be 0.2 mm, 0.5 mm, 0.9 mm, 0.7 mm, and 0.5 mm, respectively. The results suggest that it is possible to capture more anatomical structures using the transducer with an integrated amplifier due to the high detection sensitivity. The CNRs do not show uniform decreasing along the depth because of the uneven tissue surface. As shown in Figure 10d, although the wire L2 and L5 were located at similar depths under the surface, the CNR of L5 was actually much lower than that of L1. The reason is that the incident light was deflected by the tilted surface before going deeper in the tissue, resulting in low optical energy deposited on wire L5. Overall, the CNR of the PA image was improved by 10 dB using the aUST compared with that using UST, demonstrating the capability of sensitivity-enhanced PA imaging using the transducer with an integrated front-end amplifier.

## 4. Conclusions

In this work, a PZT transducer with an integrated front-end amplifier was developed to improve the detection sensitivity of PA imaging. High sensitivity is achieved by interfacing an amplifier die closely to the transducer to reduce the additional noise and provide signal amplification locally.

Acoustic characterization shows that the transducer with an integrated front-end amplifier had a fractional bandwidth of 63% and achieved a high detection sensitivity of 62.1 μV/Pa and a low NEP of 0.24 mPa/√Hz at the central frequency of 20 MHz. Imaging experiments on wire phantoms and the chicken breast tissue demonstrated that the fabricated prototype was capable of improving the CNR by 10 dB and extending the imaging depth by ~1 mm. These results imply that integrating front-end amplifier to the transducer is a promising method for improving the detection sensitivity and has the potential for enhancement of image quality and penetration depth of PA imaging.

The laser energy per pulse was adjusted to be hundreds of nJ to demonstrate that the transducer with an integrated front-end amplifier was able to obtain high contrast images at such low light fluence. It is possible to further improve the imaging depth by increasing the laser energy within the ANSI limits. The size of the transducer used in this work was not comparable to that reported previously [12,15,16], which also limits the imaging depth range. The axial resolution achieved in this work was 104 μm. Since an unfocused transducer with a relative large aperture was used for acoustic detection, the lateral resolution of the current system (1.4 mm) was predominantly limited by the size of the illumination spot. As future work, a focused transducer or a tightly focused light will be used to achieve high-resolution PA imaging. The miniature size of the amplifier die and the compact integration scheme make the proposed method also possible for endoscopic application or array-based photoacoustic tomography where the size of the element is typically confined to a sub-millimeter.

In summary, coupling the front-end amplifier directly to the transducer is capable of improving the detection sensitivity and the penetration depth of PA imaging and may open up the possibility of small structures detection, weak physiological changes monitoring, and functional parameters measurements in deep tissue.

## Figures and Tables

**Table 1:** Summary of the receiving performance.

	Specifications					
Transducers	Pulse Width (ns)	Center Frequency (MHz)	−6 dB Bandwidth (%)	SNR at 3 mm (dB)	Sensitivity at 20 MHz (μV/Pa)	NEP at 20 MHz (mPa/√Hz)
UST	99.8	19.75	41.6	50.5	4.3	0.64
aUST	101.0	19.25	40.5	62.5	62.1	0.24

## References

[1] Ntziachristos V. (2010). Going deeper than microscopy: the optical imaging frontier in biology. Nat. Methods.

[2] Zackrisson S., van de Ven S.M.W.Y., Gambhir S.S. (2014). Light In and Sound Out: Emerging Translational Strategies for Photoacoustic Imaging. Cancer Res..

[3] Meng J., Song L. (2013). Biomedical photoacoustics in China. Photoacoustics.

[4] Taruttis A., Ntziachristos V. (2015). Advances in real-time multispectral optoacoustic imaging and its applications. Nat. Photonics.

[5] Choi W., Park E.Y., Jeon S., Kim C. (2018). Clinical photoacoustic imaging platforms. Biomed. Eng. Lett..

[6] Valluru K.S., Chinni B.K., Rao N., Bhatt S., Dogra V.S. (2009). Basics and Clinical Applications of Photoacoustic Imaging. Ultrasound Clin..

[7] Xia J., Yao J., Wang L. (2014). Photoacoustic Tomography: Principles and Advances. Prog. Electromagn. Res..

[8] Steinberg I., Huland D.M., Vermesh O., Frostig H.E., Tummers W.S., Gambhir S.S. (2019). Photoacoustic clinical imaging. Photoacoustics.

[9] Zeng L., Piao Z., Huang S., Jia W., Chen Z. (2015). Label-free optical-resolution photoacoustic microscopy of superficial microvasculature using a compact visible laser diode excitation. Opt. Express.

[10] Zhang E., Laufer J.G., Pedley R.B., Beard P.C. (2009). In vivo high-resolution 3D photoacoustic imaging of superficial vascular anatomy. Phys. Med. Biol..

[11] Zhang H., Maslov K., Li M., Stoica G., Wang L. (2006). In vivo volumetric imaging of subcutaneous microvasculature by photoacoustic microscopy. Opt. Express.

[12] Stylogiannis A., Prade L., Buehler A., Aguirre J., Sergiadis G., Ntziachristos V. (2018). Continuous wave laser diodes enable fast optoacoustic imaging. Photoacoustics.

[13] Schwarz M., Aguirre J., Buehler A., Omar M., Ntziachristos V. Visualization of the microcirculatory network in skin by high frequency optoacoustic mesoscopy. Proceedings of the Opto-Acoustic Methods and Applications in Biophotonics II.

[14] Wang X., Pang Y., Ku G., Xie X., Stoica G., Wang L. (2003). Noninvasive laser-induced photoacoustic tomography for structural and functional in vivo imaging of the brain. Nat. Biotechnol..

[15] Qin W., Jin T., Guo H., Xi L. (2018). Large-field-of-view optical resolution photoacoustic microscopy. Opt. Express.

[16] Aguirre J., Schwarz M., Soliman D., Buehler A., Omar M., Ntziachristos V. (2014). Broadband mesoscopic optoacoustic tomography reveals skin layers. Opt. Lett..

[17] Jansen K., Van Soest G., Van Der Steen A.F.W. (2014). Intravascular photoacoustic imaging: a new tool for vulnerable plaque identification. Ultrason. Med. Biol..

[18] Hui J., Cao Y., Zhang Y., Kole A., Wang P., Yu G., Eakins G., Sturek M., Chen W., Cheng J.-X. (2017). Real-time intravascular photoacoustic-ultrasound imaging of lipid-laden plaque in human coronary artery at 16 frames per second. Sci. Rep..

[19] Zhang J., Yang S., Ji X., Zhou Q., Xing D. (2014). Characterization of Lipid-Rich Aortic Plaques by Intravascular Photoacoustic Tomography Ex Vivo and In Vivo Validation in a Rabbit Atherosclerosis Model With Histologic Correlation. J. Am. Coll. Cardiol..

[20] Bendinger A.L., Glowa C., Peter J., Karger C.P. (2018). Photoacoustic imaging to assess pixel-based sO(2) distributions in experimental prostate tumors. J. Biomed. Opt..

[21] Wood C., Harutyunyan K., De La Cerda J., Kaffes C., Millward N.Z., Shanmugavelandy S., Konopleva M., Bouchard R. (2018). Assessment of blood oxygen saturation using spectroscopic photoacoustic imaging as a biomarker for disease progression in a small-animal leukemia model. Medical Imaging 2018: Ultrasonic Imaging And Tomography.

[22] Zhang H.F., Maslov K., Stoica G., Wang L.V. (2006). Functional photoacoustic microscopy for high-resolution and noninvasive in vivo imaging. Nat. Biotechnol..

[23] Cao R., Li J., Ning B., Sun N., Wang T., Zuo Z., Hu S. (2017). Functional and oxygen-metabolic photoacoustic microscopy of the awake mouse brain. Neuroimage.

[24] Larina I.V., Larin K.V., Esenaliev R.O. (2005). Real-time optoacoustic monitoring of temperature in tissues. J. Phys. D Appl. Phys..

[25] Yao J., Ke H., Tai S., Zhou Y., Wang L. (2013). Absolute photoacoustic thermometry in deep tissue. Opt. Lett..

[26] Landa F.J.O., Dean-Ben X.L., Sroka R., Razansky D. (2018). Four-dimensional optoacoustic temperature mapping in laser-induced thermotherapy. Photons Plus Ultrasound: Imaging And Sensing.

[27] Nie L., Chen M., Sun X., Rong P., Zheng N., Chen X. (2014). Palladium nanosheets as highly stable and effective contrast agents for in vivo photoacoustic molecular imaging. Nanoscale.

[28] Paproski R.J., Forbrich A., Harrison T., Hitt M., Zemp R.J. (2011). Photoacoustic imaging of gene expression using tyrosinase as a reporter gene. Photons Plus Ultrasound: Imaging and Sensing.

[29] Paproski R.J., Heinmiller A., Wachowicz K., Zemp R.J. (2014). Multi-wavelength photoacoustic imaging of inducible tyrosinase reporter gene expression in xenograft tumors. Sci. Rep..

[30] Weber J., Beard P.C., Bohndiek S.E. (2016). Contrast agents for molecular photoacoustic imaging. Nat. Methods.

[31] Liu W., Shcherbakova D.M., Kurupassery N., Li Y., Zhou Q.F., Verkhushaz V.V., Yao J.J. (2018). Quad-mode functional and molecular photoacoustic microscopy. Sci. Rep..

[32] Ji X.R., Xiong K.D., Yang S.H., Xing D. (2015). Intravascular confocal photoacoustic endoscope with dual-element ultrasonic transducer. Opt. Express.

[33] Galanzha E.I., Shashkov E.V., Kelly T., Kim J.W., Yang L., Zharov V.P. (2009). In vivo magnetic enrichment and multiplex photoacoustic detection of circulating tumour cells. Nat. Nanotechnol..

[34] Mehrmohammadi M., Yoon S.J., Yeager D., Emelianov S.Y. (2013). Photoacoustic Imaging for Cancer Detection and Staging. Curr. Mol. Imaging.

[35] Beard P. (2011). Biomedical photoacoustic imaging. Interface Focus.

[36] Rosenthal A., Razansky D., Ntziachristos V. (2011). High-sensitivity compact ultrasonic detector based on a pi-phase-shifted fiber Bragg grating. Opt. Lett..

[37] Hoskins P., thrush a., Martin K., Whittingham T. (2003). Diagnostic ultrasound: physics and equipment.

[38] Yao J., Wang L.V. (2014). Sensitivity of photoacoustic microscopy. Photoacoustics.

[39] White I.J., Dederich H.D. (2007). American National Standard for the Safe Use of Lasers, ANSI Standard Z 136.1 2007.

[40] Chan J., Zheng Z., Bell K., Le M., Reza P.H., Yeow J.T.W. (2019). Photoacoustic Imaging with Capacitive Micromachined Ultrasound Transducers: Principles and Developments. Sensors.

[41] Lutzweiler C., Razansky D. (2013). Optoacoustic Imaging and Tomography: Reconstruction Approaches and Outstanding Challenges in Image Performance and Quantification. Sensors.

[42] Chen C., Raghunathan S.B., Yu Z., Shabanimotlagh M., Chen Z., Chang Z., Blaak S., Prins C., Ponte J., Noothout E. (2016). A Prototype PZT Matrix Transducer With Low-Power Integrated Receive ASIC for 3-D Transesophageal Echocardiography. IEEE Trans. Ultrason. Ferroelectr. Freq. Control.

[43] Wildes D., Lee W., Haider B., Cogan S., Sundaresan K., Mills D.M., Yetter C., Hart P.H., Haun C.R., Concepcion M. (2016). 4-D ICE: A 2-D Array Transducer With Integrated ASIC in a 10-Fr Catheter for Real-Time 3-D Intracardiac Echocardiography. IEEE Trans. Ultrason. Ferroelectr. Freq. Control.

[44] Moini A., Nikoozadeh A., Choe J.W., Chang C., Stephens D.N., Sahn D.J., Khuri-Yakub P.T. Fully Integrated 2D CMUT Ring Arrays for Endoscopic Ultrasound. Proceedings of the 2016 IEEE International Ultrasonics Symposium (IUS).

[45] Jeon S., Kim J., Lee D., Baik J.W., Kim C. (2019). Review on practical photoacoustic microscopy. Photoacoustics.

[46] Yang C., Jian X., Zhu X., Lv J., Han Z., Sergiadis G., Cui Y. Highly sensitive PZT transducer with integrated miniature amplifier for photoacoustic imaging. Proceedings of the 2019 IEEE International Ultrasonics Symposium (IUS).

[47] Hicks B., Erickson B. (2018). Bias-T Design Considerations for LWA. LWA Memo 135. http://www.ece.vt.edu/swe/lwa.

[48] Chen B., Chu F., Liu X., Li Y., Rong J., Jiang H. (2013). AlN-based piezoelectric micromachined ultrasonic transducer for photoacoustic imaging. Appl. Phys. Lett..

[49] Wygant I.O., Zhuang X., Yeh D.T., Oralkan O., Ergun A.S., Karaman M., Khuri-Yakub B.T. (2008). Integration of 2D CMUT arrays with front-end electronics for volumetric ultrasound imaging. IEEE Trans. Ultrason. Ferroelectr. Freq. Control.

[50] Vallet M., Varray F., Boutet J., Dinten J.M., Caliano G., Savoia A.S., Vray D. (2017). Quantitative comparison of PZT and CMUT probes for photoacoustic imaging: Experimental validation. Photoacoustics.

